# Technique-Specific Load–Velocity Profiling and Prediction Equation for the Back Squat in Elite Portuguese Rugby Players

**DOI:** 10.3390/sports14070298

**Published:** 2026-07-13

**Authors:** Gonçalo Rendeiro-Pinho, André Sousa, Antonio P. Veloso, Javier Riscart-López

**Affiliations:** 1 Performance Sports Centre, Go Athlete, Calçada Rio, Campo de Futebol 60, 1495-113 Algés, Portugal; goncalopinho@gmail.com (G.R.-P.); andrepereirasousa@gmail.com (A.S.); 2 Faculty of Human Kinetics, University of Lisbon, 1649-004 Lisbon, Portugal; 3 Centro Interdisciplinar de Estudo da Performance Humana (CIPER), Faculty of Human Kinetics, Lisbon University, 1495-751 Oeiras, Portugal; apveloso@fmh.ulisboa.pt; 4 Faculty of Medicine, University of Cádiz, 11003 Cádiz, Spain; 5 Salud, Deporte e Inflamación Research Group, Biomedical Research and Innovation Institute of Cádiz (INiBICA), Puerta del Mar University Hospital, University of Cádiz, Plaza Fragela, s/n, 11003 Cádiz, Spain

**Keywords:** velocity-based training, squat, rugby, equation, exercise

## Abstract

This study examined whether barbell velocity can accurately predict relative load during a free-weight parallel squat performed with a deliberate pause between the eccentric and concentric phases in elite Portuguese rugby players. A repeated-measures design was used, in which 25 athletes completed an incremental loading protocol up to their one-repetition maximum (1-RM) on two occasions separated by four weeks. The relationship between mean propulsive velocity (MPV) and %1-RM was analyzed using polynomial regression, and a second-order prediction equation was developed. The model showed a very strong association between MPV and %1-RM (R^2^ = 0.95) with a standard error of estimate of 6.27 %1-RM, indicating good practical accuracy. Test–retest analyses demonstrated high stability of the load–velocity relationship (mean velocity difference: −0.01 ± 0.02 m·s^−1^). Compared with values reported in other athletic populations, first-division rugby players displayed distinct load–velocity profiles across the full loading spectrum, underscoring the influence of execution technique and population characteristics on load–velocity behavior. These findings support the use of individualized velocity-based prescriptions rather than generalized equations. In conclusion, this study provides a technique- and population-specific prediction equation for the paused parallel back squat, offering a novel and practical tool for regulating training intensity in elite rugby players.

## 1. Introduction

Rugby union is a collision-based team sport in which players are repeatedly exposed to short bouts of high-intensity actions—such as sprinting, tackling, rapid accelerations and decelerations, and high-speed running—interspersed with brief periods of lower-intensity activity and partial recovery [1]. These repeated neuromuscular and mechanical demands require well-developed strength and power capacities to tolerate contact situations, maintain performance across repeated efforts, and support key match actions [2,3,4]. Consequently, structured strength training is a central component of physical preparation in rugby, aiming to enhance maximal force production, power output, and sprint performance [5,6]. Among resistance exercises, the back squat is considered a fundamental movement pattern for developing lower-limb musculature, contributing to injury risk reduction and improving overall athletic performance [7,8].

A wide range of squat variations has been described previously, differing in joint range of motion, depth, bar position and execution strategy [9,10,11]. Two of the main technical determinants are squatting depth [10,12,13,14] and whether the transition between the eccentric and concentric phases is performed in a continuous (ballistic) manner or with a deliberate pause [15]. Performing a parallel back squat—defined as descending until the inguinal crease is aligned with the top of the knee [10]—combined with a standardized pause at the bottom position allows practitioners to closely reproduce the same eccentric range of motion across repetitions and sessions. This approach reduces intra-subject variability and, consequently, decreases measurement error, leading to more reliable isoinertial strength assessments and more robust interpretation of testing and training outcomes by professionals in sport science [15].

Despite the central role of the squat in rugby strength programs, one of the main practical challenges is the accurate and objective quantification of the actual load imposed during resistance training [16]. Several frameworks have been proposed to guide resistance training prescription and to describe how manipulating variables such as volume, intensity, rest intervals or contraction mode influences neuromuscular and molecular adaptations [17,18]. Traditionally, training load has typically been determined using a percentage of the one-repetition maximum (1-RM), using %1-RM to define submaximal loads [19]. However, routine assessment of 1-RM presents important limitations, including time constraints, fatigue, potential injury risk and sensitivity to day-to-day fluctuations in performance [16,20]. An alternative strategy is to prescribe intensity based on the maximum number of repetitions (MNR) that can be performed with a given load, although this method is also affected by considerable variability and does not provide a sufficiently precise estimate of relative intensity [21]. These limitations highlight the need for alternative approaches capable of accurately estimating %1-RM—and, by extension, 1-RM—while minimizing the drawbacks associated with traditional methods.

In recent years, advances in sport technology have enabled the development of velocity-based methods, in which barbell movement velocity is typically measured with linear position transducers, used to predict 1-RM, and determine relative load [16,22,23,24,25,26,27]. The core benefit of this velocity-based method is that, when the concentric phase is performed with maximal intended effort, the relative load (%1-RM) can be determined in real time with a high degree of precision [15,16,23,24,25,26,27,28,29,30]. This allows daily adjustment of the absolute load (kg) to match the target %1-RM and allows maximal-strength changes to be monitored across the training program without repeated maximal testing [29,30,31]. The feasibility of this strategy is supported by numerous studies reporting strong and stable relationships between movement velocity and %1-RM in several resistance exercises, including different squat variants [10,15,23,25,26,27,28,29,30,31,32].

An important limitation within the subset of studies focused on velocity-based load estimation is that many load–velocity equations have been developed using Smith-machine (Peroga Fitness, Murcia, Spain) protocols, whereas fewer investigations have examined this relationship under strictly standardized free-weight conditions [23,27,32]. This distinction is relevant because the load–velocity profile appears to be both exercise-specific and mode-specific, and free-weight squats are generally preferred by athletes and practitioners. Moreover, previous research suggests that free-weight squats may elicit greater neuromuscular and functional adaptations compared with Smith-machine squats [33,34]. Taken together, the available evidence indicates that the validity of using load–velocity relationships to prescribe and monitor intensity in the parallel back squat depends on several factors, including whether the exercise is performed with free weights or in a Smith machine, the depth of the squat, the presence or absence of a pause between eccentric and concentric phases, and the characteristics of the population studied (e.g., team-sport athletes, recreational lifters).

In this context, there is a lack of specific evidence regarding the use of barbell velocity to estimate relative load in free-weight parallel back squats performed with a pause in elite rugby players. Therefore, the present study aimed to examine the validity of using barbell velocity to estimate relative load (1RM) in a free-weight parallel squat with a pause between the eccentric and concentric phases (SQ) in first-division Portuguese rugby players. Based on the previous literature showing exercise- and population-specific load–velocity profiles, it was hypothesized that mean propulsive velocity would display a strong and predictable association with %1-RM in this cohort, allowing accurate estimation of relative load from velocity measurements.

## 2. Materials and Methods

### 2.1. Study Design

A cross-sectional design was implemented to develop an equation capable of estimating the %1-RM in the paused parallel squat (SQ) for first-division Portuguese rugby players. To achieve this, a linear regression model was constructed using mean propulsive velocity (MPV) obtained during submaximal loads as the predictor of the actual %1-RM. Each athlete completed an incremental free-weight squat assessment in which the load was progressively increased until reaching their true 1-RM, allowing the full load–velocity profile to be established. All testing procedures were carried out in a controlled laboratory environment under the supervision of experienced researchers. Sessions were scheduled at the same time of day (±1 h) and conducted under stable environmental conditions (approximately 20 °C and 60% humidity) to minimize external variability. Throughout the protocol, players were instructed to perform every concentric action with maximal intent and were provided with strong verbal encouragement to ensure consistent maximal effort during all repetitions.

### 2.2. Subjects

A total of 25 rugby players from of Portuguese first division volunteered to participate in this study. All rugby players had at least 5 years of strength-training experience and were able to perform the SQ with proper technique. The participants’ characteristics are presented in Table 1. Once informed about the purpose, procedures, and potential risks of the investigation, data confidentiality and their right to withdraw from the study without consequences, all subjects gave their voluntary written consent to participate. The present investigation was approved in 2022 (protocol code: UCA/7.22) by the University of Cadiz Ethics Committee for Research Involving Human Subjects and conducted in accordance with the Declaration of Helsinki. The medical examination revealed no physical limitations, health issues, or musculoskeletal injuries that could interfere with training. None of the participants were taking drugs, medications, or dietary supplements.

### 2.3. Testing Procedures

Players reported to the laboratory after completing a 48-h rest period to ensure adequate recovery before testing. Upon arrival, anthropometric data were collected, after which the physical assessment began. The evaluation consisted of a progressive loading protocol in the free-weight parallel squat with a pause (SQ). The squat started from an upright position with the hips and knees fully extended, the feet set at approximately shoulder-width apart, and the barbell resting across the upper back at acromion height. Foot placement and stance width were individually standardized and reproduced identically throughout all attempts. Participants were required to maintain full foot contact with the ground and keep the barbell securely on their shoulders during both the eccentric and concentric phases; any deviation resulted in the repetition being invalidated and repeated. Each athlete descended in a controlled manner until the inguinal crease aligned with the top of the knee, corresponding to the parallel squat depth. To ensure consistent range of motion and to impose a deliberate pause between the eccentric and concentric phases, two adjustable telescopic bar supports (precision ±1 cm) were positioned on either side of the athlete. These supports were set at each individual’s parallel depth and served as a reference point to standardize the pause. After lowering the barbell to the prescribed velocity (0.45–0.65 m·s^−1^), athletes briefly settled the bar on the supports for a 2-s pause—maintaining contact without fully unloading—and then executed the concentric phase with maximal intent.

The warm-up protocol included 5 min of light jogging, followed by 5 min of joint mobility work. Athletes then performed two preparatory sets of squats: eight repetitions with 20 kg and six repetitions with 30 kg, separated by 3-min rest intervals. The testing began with 30 kg, and load increments of 15 kg were applied until the MPV dropped below 0.70 m·s^−1^. Subsequent increases were smaller (5 to 2.5 kg) to refine the load–velocity profile. The heaviest load that each athlete could lift with proper technique (±2.5 kg) was recorded as their 1-RM. The number of repetitions varied according to intensity: three repetitions for loads ≤ 50% 1-RM, two repetitions for 50–70% 1-RM, and a single repetition for loads ≥ 80% 1-RM.

### 2.4. Measurement Equipment and Data Analysis

Barbell velocity was recorded using a linear velocity transducer (T-Force System, Ergotech, Murcia, Spain), which operates as an optical rotary encoder sampling at 1000 Hz. The system was calibrated before testing following the manufacturer’s procedure, ensuring correct cable displacement and zero-offset adjustment. The device was attached perpendicularly to the barbell to minimize angular error. Mean propulsive velocity (MPV) was extracted for each repetition, defined as the portion of the concentric phase during which acceleration exceeded gravitational acceleration [35]. The T-Force has shown high validity and reliability for measuring barbell velocity in resistance-training tasks (typical error < 0.01 m·s^−1^; CV < 1%) [36].

### 2.5. Data Analysis

An a priori power analysis was conducted using G*Power (G*Power software version 3.1; Heinrich Heine University Düsseldorf, Düsseldorf, Germany) for a regression model examining the relationship between MPV and %1-RM. Assuming a large expected effect size based on previous literature (R^2^ ≈ 0.80–0.90), an alpha level of 0.05, and the final sample size (*n* = 25), the resulting statistical power exceeded 0.99, indicating that the study was adequately powered to detect the observed association.

Standard statistical procedures were applied to compute the mean, standard deviation (SD), coefficient of variation (CV), coefficient of determination (R^2^), standard error of the estimate (SEE), 95% confidence intervals (CI), and Pearson correlation coefficients (r).

Data normality for all continuous variables was assessed using the Shapiro–Wilk test, and homogeneity of variance was examined using Levene’s test, with no assumption departures detected. The relationship between load (%1-RM) and velocity was examined using second-order polynomial models, which provided slightly better fits than linear functions. A one-way ANOVA was used to identify differences between subject subgroups for age, body mass, RSR, mean test velocity, MPV at each %1-RM, and V-1-RM. When significant effects were detected, Scheffé post hoc tests were applied. Eta-squared ($\eta_{p}^{2}$) was calculated as the ratio of between-group to total sum of squares and reported as the ANOVA effect size. All analyses were conducted using SPSS (IBM SPSS v23.0, Chicago, IL, USA). Statistical significance was set at *p* ≤ 0.05.

## 3. Results

### 3.1. Squat Performance

1-RM strength for the SQ was 150.2 ± 28.9 kg (i.e., 1.60 ± 0.40 kg normalized per kg of body mass). Subjects performed a total of 7.6 ± 1.5 increasing loads up to the 1-RM in the progressive loading test.

### 3.2. Load–Velocity Relationship

**Figure 1:**
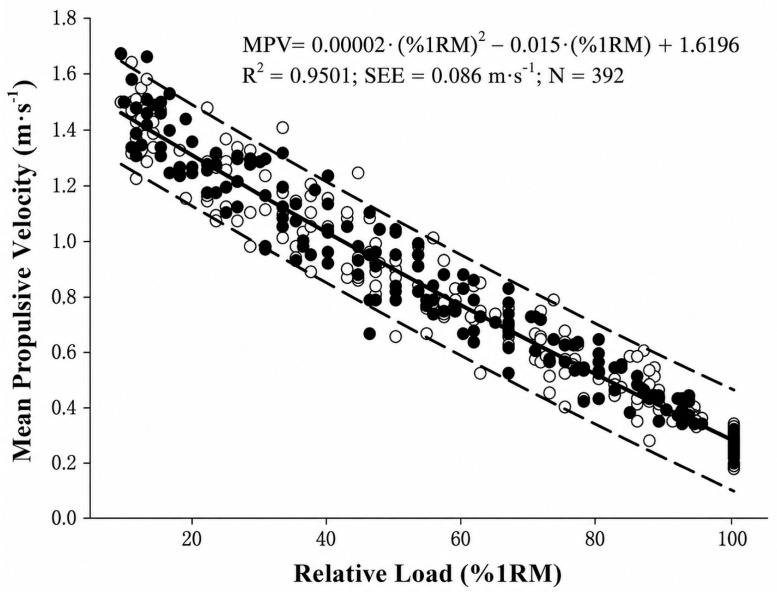
The relationship between relative load (%1-RM) and mean propulsive velocity (MPV) was derived from 392 raw data points obtained across the incremental tests. Raw %1-RM–MPV values from the progressive loading protocol performed by 25 participants are shown for the Pre-test (dashed line) and Post-test (solid line). The thick line represents the fitted curve, and the thin lines denote the 95% prediction limits. SEE: standard error of the estimate.

The analyzed velocity variables were plotted against %1-RM, yielding 392 raw load–velocity data pairs (Figure 1). Plotting mean propulsive velocity (MPV) against %1-RM and fitting a second-order polynomial to all observations revealed a very strong association between both variables (R^2^ = 0.95; Figure 1). The second-order polynomial derived from the relationship between relative load and MPV is shown in Figure 1. The mean coefficient of determination for the individual fits was R^2^ = 0.991 ± 0.013 (95% CI: 0.987–0.994; CV = 1.28%).

### 3.3. Estimating %1-RM from Velocity Measures

Given that the practical objective is to infer relative load (%1-RM) from velocity data, treating velocity as the independent variable allows the derivation of a regression equation capable of estimating %1-RM from mean propulsive velocity (MPV, m·s^−1^):

%1-RM = 16.035 · MPV^2^ − 102.23 · MPV + 129.59 (R^2^ = 0.954; SEE = 6.27% 1RM)

The %1RM associated with each MPV was obtained from these polynomial fits, from 20% 1-RM onwards, in 5% increments (Table 2). The MPV at which 1-RM was attained was: 0.30 ± 0.03 m·s^−1^.

### 3.4. Stability in the Load–Velocity Relationship

A total of 51 participants completed a retest after 6 weeks of training to examine potential changes in the MPV associated with each relative load following modifications in maximal strength. From T1 to T2, mean 1-RM increased by 5.3 ± 3.2% (from 150.2 ± 28.9 kg to 158.2 ± 26.3 kg). Nevertheless, the difference in mean test velocity was only of −0.01 ± 0.05 m·s^−1^ or, when expressed as absolute values, of 0.01 ± 0.02 m·s^−1^ (Table 3).

## 4. Discussion

The findings of this investigation support the initial hypothesis: the mean propulsive velocity produced against a given absolute load in the squat can serve as a highly accurate indicator of the relative load (%1-RM) being lifted by first-division Portuguese rugby players, as long as each repetition is executed with maximal concentric intent. A key practical implication of this result is the ability to determine the actual training load in real time simply by monitoring bar velocity. This enables coaches to prescribe and regulate resistance training based on movement speed rather than relying on predetermined percentages of 1-RM or estimates derived from maximum-repetition schemes. Such an approach enhances the precision of load adjustments and allows for more individualized and responsive training prescriptions.

The MPV values examined in this study demonstrated a very strong association with the corresponding %1-RM (R^2^ = 0.95) (Figure 1). Although the SEE of 6.27%1RM indicates that some degree of uncertainty is inherent to velocity-based load estimation, this magnitude of error is comparable to values reported in previous studies and may be acceptable for practical intensity prescription in applied sport settings. Nonetheless, the prediction error should be considered when interpreting the model’s usefulness, particularly in contexts where small variations in load may have meaningful training implications. These findings align with previous research showing that movement velocity is closely linked to relative load in the squat exercise [10,15,23,32]. Several studies have proposed prediction models based on MPV to estimate %1-RM, and the present results reinforce the usefulness of this approach. Although alternative modelling approaches, such as linear regression or mixed-effects models, have been proposed, a second-order polynomial regression was selected because it provided a better fit to the empirical MPV–%1-RM relationship than a linear model, as reflected by a higher coefficient of determination (R^2^ = 0.95) and a lower standard error of estimate (SEE = 6.27 %1-RM). Mixed-effects models were not used because the primary objective was to derive a practical, population-specific prediction equation rather than to model interindividual variability. Prior studies have emphasized that mean propulsive velocity should be favored over mean concentric velocity because it more accurately reflects an individual’s neuromuscular capacity, particularly when working with light to moderate loads [15,16,35]. Additionally, earlier evidence suggests that factors such as training background or the specific characteristics of the athletic population may influence the accuracy of load–velocity equations [37]. The current investigation expands this knowledge by demonstrating that these principles also apply to the paused parallel squat in elite Portuguese rugby players, providing population-specific data that had not been previously documented.

Previous investigations have reported that the mean velocities associated with the actual 1-RM in different squat variations typically fall around 0.23 ± 0.05, 0.10 ± 0.04, and 0.14 ± 0.05 m·s^−1^, values that are noticeably lower than those obtained in the present study. This observation reinforces the idea that the velocity at which maximal strength is expressed is influenced both by the specific squat technique employed and by the characteristics of the population being assessed. As illustrated in Table 1, the MPV corresponding to each relative load from 25% and 100% 1-RM in the paused parallel squat differs from previously published data. Earlier studies consistently reported higher MPV values across most submaximal intensities—typically 0.07 to 0.09 m·s^−1^ faster from 25% to 70% 1-RM, and similarly elevated values between 70% and 80% 1-RM. In contrast, the velocities recorded at the heaviest loads were comparable across studies, with the 1-RM reached at nearly identical speeds (SQ: 0.33 ± 0.06 vs. 0.30 ± 0.03 m·s^−1^). Overall, prior research shows higher bar velocities at almost all loads up to 95% 1-RM compared with the present findings. Several methodological factors may explain these discrepancies, including the use of different velocity metrics (MV vs. MPV), the execution mode (free-weight vs. Smith machine), variations in squat depth, and whether the movement was performed ballistically or with a deliberate pause. Another important consideration is the nature of the sample used in this study. The participants were elite rugby players competing in the Portuguese first division, a population with substantial strength and power demands. Such athletes may exhibit a greater strength deficit, defined as the proportion of maximal force capacity that is not expressed during a specific motor task [38], when compared with recreationally active individuals or athletes from other sports such as soccer. In this regard, elite rugby players display neuromuscular characteristics shaped by the collision-based, high-intensity intermittent nature of the sport, including greater maximal strength, enhanced eccentric force capacity, and a high tolerance to repeated high-force actions. These attributes may influence barbell velocity across the loading spectrum and help explain the distinct load–velocity profiles observed in this population compared with other athletic groups. Although the present study did not include statistical comparisons between different populations, previously published load–velocity data are referenced to illustrate that MPV values at equivalent %1RM differ across cohorts and execution styles. These comparisons are descriptive and intended solely to contextualize the findings, reinforcing the need for exercise- and population-specific prediction equations rather than generalized models.

The interpretation of the present findings should be made cautiously, as the strength of the associations reported may be influenced by the statistical strategy employed. Although the model demonstrated high explanatory capacity (R^2^ = 0.95) and acceptable prediction error (SEE = 6.27 %1-RM), additional procedures such as cross-validation, residual diagnostics, and external validation using independent samples were not feasible with the present dataset. Given these limitations, the practical use of the proposed prediction equation should be approached with caution. Although the model performed well within the present sample, its applicability beyond this specific cohort remains uncertain; therefore, the equation should not be applied in other populations or contexts until it has been validated in independent samples using appropriate cross-validation and external validation procedures. These steps are essential to confirm the generalizability of the model and ensure that its estimates are reliable across different athlete profiles and execution strategies.

Future research should incorporate these approaches to further assess generalizability and minimize the risk of model overfitting. Several methodological factors can influence the magnitude of load–velocity associations. While most previous investigations with similar objectives have relied primarily on between-subject analyses, more recent methodological recommendations emphasize the value of within-subject approaches to better capture individual responses [39,40]. In this context, it is important to note that although the relationship between force, load, and movement velocity has long been recognized in the scientific literature, the present study focuses specifically on the empirical load–velocity relationship obtained during a multi-joint isoinertial exercise. This relationship reflects the mechanical behavior of the athlete–exercise system under external loading conditions rather than the intrinsic contractile properties described in classical muscle models. Barbell velocity depends on the net force applied. When maximal strength increases, the athlete can apply proportionally more force, but the mass corresponding to a given %1-RM also increases. As a result, the ratio between applied force and mass remains similar, producing nearly identical velocity profiles at the same relative intensity. This explains why an athlete who moves 80% 1-RM at ~0.53 m·s^−1^ before training will typically move at the “new 80% 1-RM” at ~0.53 m·s^−1^ after increasing strength. What changes is the absolute load (kg), not the characteristic velocity associated with that %1-RM. This stability is precisely what allows velocity-based profiling to estimate relative load reliably. Performance improvements in velocity-based training can therefore manifest in two complementary ways: either by lifting a greater absolute load at the same velocity, reflecting an increase in maximal strength, or by lifting the same absolute load at a higher velocity, which occurs when that load represents a lower percentage of the updated 1-RM. Together, these mechanisms illustrate how strength gains translate mechanically in the context of velocity-based training. To further clarify the robustness and applicability of velocity-based load prediction models, future studies should validate the proposed equation in independent samples, across different strength levels and sport disciplines, and under alternative execution conditions (e.g., without a pause, different squat depths). Investigations incorporating larger and more diverse cohorts, as well as advanced validation procedures, will be essential to confirm the generalizability of the present findings.

In summary, our results demonstrate that movement velocity is a valid and precise indicator for prescribing relative load (%1-RM) in the squat for first-division Portuguese rugby players. However, when applying these findings in testing or training environments, practitioners should recognize that the velocity associated with each percentage of 1-RM may vary depending on several factors: the specific velocity metric used (MV vs. MPV), the technical characteristics of the squat (free-weight vs. Smith machine, depth of descent, and whether a pause or ballistic transition is used), and the population being assessed (team-sport athletes, recreational lifters, etc.). These considerations are essential for ensuring accurate load prescription and individualized monitoring.

## 5. Conclusions

The present findings indicate that mean propulsive velocity (MPV) is a highly accurate indicator for estimating relative load (–1RM) in the SQ among first-division Portuguese rugby players. However, the velocity associated with a given %1-RM is not universal. Different squat execution strategies—such as free-weight versus Smith machine performance, variations in depth, or the use of a pause versus a continuous (ballistic) transition—produce distinct velocity profiles at each relative load.

In contrast, when comparing populations, our data suggest that rugby players with high strength levels display velocity–load relationships that are broadly similar to those reported in other athletic groups when performing the same squat variation. This supports the relevance of individualized load–velocity profiling for each specific execution technique, although population-specific equations should be interpreted cautiously, particularly in the absence of external validation.

Accordingly, accurate monitoring of training intensity through movement velocity requires tailoring the load–velocity profile to the exact exercise technique employed. While the present equation may offer practical utility for practitioners working with similar athletes, its applicability beyond this cohort remains to be confirmed. Strength and conditioning coaches may integrate the proposed model into routine monitoring by using MPV to adjust daily training loads, track neuromuscular readiness, and prescribe intensity without relying on maximal or repetition-to-failure testing, while remaining aware of the inherent prediction error and the need for context-specific interpretation.

## 6. Practical Applications

The present findings show that mean propulsive velocity (MPV) is a highly precise indicator for estimating relative load (%1-RM) in the squat for first-division Portuguese rugby players. These results reinforce the value of velocity-based training as a practical alternative to traditional methods, allowing coaches to prescribe and adjust loads without relying on 1-RM or maximum-repetition testing. This approach offers a more responsive and individualized way to monitor strength performance during training.

It is also important to recognize that athletes from this population exhibit velocity–load profiles that differ from those reported in other groups. High-performance rugby players tend to produce distinct bar-speed values at equivalent %1-RM, likely due to the specific strength demands of their sport. Consequently, load–velocity profiles should be individualized for each athlete and tailored to the exact squat execution technique used, rather than applying generalized equations across different populations or movement variations.

By establishing personalized velocity–load relationships, practitioners can more accurately regulate training intensity and ensure that resistance-training prescriptions reflect the true neuromuscular capabilities of each player.

## Figures and Tables

**Table 1:** Age, height, body mass and strength level of the Rugby Players.

	Rugby Players
Age (yr)	24.9 ± 3.2
Height (cm)	180.4 ± 4.2
Body mass (kg)	93.7 ± 14.9
RSR	1.60 ± 0.40

Data are mean ± SD; *n* = 25; RSR: relative strength ratio (1-RM strength/body mass ratio).

**Table 2:** Mean propulsive velocity attained with each relative load (%1-RM) in SQ.

Relative Load (%1-RM)	MPV	MPV 95% CI
20	1.36 ± 0.09	1.32–1.37
25	1.28 ± 0.09	1.25–1.30
30	1.20 ± 0.09	1.17–1.22
35	1.12 ± 0.09	1.09–1.14
40	1.05 ± 0.09	1.02–1.07
45	0.98 ± 0.09	0.96–1.00
50	0.91 ± 0.09	0.89–0.94
55	0.84 ± 0.08	0.82–0.86
60	0.77 ± 0.08	0.75–0.79
65	0.71 ± 0.08	0.69–0.74
70	0.65 ± 0.07	0.63–0.67
75	0.59 ± 0.07	0.57–0.61
80	0.53 ± 0.06	0.51–0.54
85	0.47 ± 0.06	0.45–0.48
90	0.41 ± 0.05	0.39–0.43
95	0.36 ± 0.04	0.35–0.38
100	0.30 ± 0.03	0.29–0.32

Data are mean ± SD (*n* = 51). SQ: Squat; MPV: mean propulsive velocity; CI: confidence interval.

**Table 3:** Modelled changes in mean propulsive velocity (m·s^−1^) achieved at each relative load, based on pooled data (T1 + T2), the initial assessment (T1), and the retest (T2) following 6 weeks of training in the squat exercise.

Relative Load (%1-RM)	General MPV	T1 MPV	T2 MPV	Difference
20	1.36 ± 0.09	1.36 ± 0.09	1.36 ± 0.09	0.00
25	1.28 ± 0.09	1.26 ± 0.09	1.26 ± 0.09	0.02
30	1.20 ± 0.09	1.20 ± 0.08	1.20 ± 0.09	0.00
35	1.12 ± 0.09	1.12 ± 0.08	1.12 ± 0.09	0.00
40	1.05 ± 0.09	1.05 ± 0.09	1.05 ± 0.09	0.00
45	0.98 ± 0.09	0.98 ± 0.09	0.98 ± 0.09	0.00
50	0.91 ± 0.09	0.91 ± 0.09	0.91 ± 0.09	0.00
55	0.84 ± 0.08	0.84 ± 0.08	0.84 ± 0.08	0.00
60	0.77 ± 0.08	0.78 ± 0.08	0.77 ± 0.08	0.01
65	0.71 ± 0.08	0.71 ± 0.08	0.71 ± 0.08	0.00
70	0.65 ± 0.07	0.65 ± 0.07	0.65 ± 0.07	0.00
75	0.59 ± 0.07	0.59 ± 0.07	0.59 ± 0.07	0.00
80	0.53 ± 0.06	0.53 ± 0.06	0.53 ± 0.06	0.00
85	0.47 ± 0.06	0.47 ± 0.06	0.47 ± 0.06	0.00
90	0.41 ± 0.05	0.42 ± 0.05	0.41 ± 0.05	0.01
95	0.36 ± 0.04	0.36 ± 0.04	0.35 ± 0.04	0.01
100	0.30 ± 0.03	0.31 ± 0.03	0.30 ± 0.03	0.01

Data are mean ± SD (*n* = 51). MPV: mean propulsive velocity.

## Data Availability

The data that supports the results of this study are available from the corresponding author upon reasonable request, as they cannot be shared publicly due to ethical restrictions.
